# The interaction effect of aerobic exercise and vitamin D supplementation on inflammatory factors, anti-inflammatory proteins, and lung function in male smokers: a randomized controlled trial

**DOI:** 10.1186/s13102-021-00333-w

**Published:** 2021-08-30

**Authors:** Leila Nikniaz, Morteza Ghojazadeh, Hooman Nateghian, Zeinab Nikniaz, Mahdieh Abbasalizad Farhangi, Hadi Pourmanaf

**Affiliations:** 1 Tabriz Health Services Management Research Center, Health Management and Safety Promotion Research Institute, Tabriz University of Medical Sciences, Tabriz, Iran; 2 Research Center for Evidence-Based Medicine, Iranian EBM Centre: A Joanna Briggs Institute Affiliated Group, Tabriz University of Medical Sciences, Tabriz, Iran; 3 Student Research Committee, Tabriz University of Medical Sciences, Tabriz, Iran; 4 Liver and Gastrointestinal Diseases Research Center, Tabriz University of Medical Sciences, Tabriz, Iran; 5 Drug Applied Research Center, Tabriz University of Medical Sciences, Tabriz, Iran; 6 Faculty of Physical Education and Sport Sciences, University of Tehran, Tehran, Iran

**Keywords:** Aerobic exercise, CC16, Vitamin D, Inflammation, SP-D, Lung function, Smoker

## Abstract

**Background:**

This study aimed to investigate the interaction effect of aerobic exercise and vitamin D supplementation on inflammation (TNF-α, IL-6, CC16, SP-D, and CC16/SP-D ratio) and lung function (FEV_1_, FVC, and FEV_1_/FVC ratio) in male smokers.

**Methods:**

After applying inclusion criteria, a total of 40 healthy male smokers were recruited in this study. The participants were randomly divided into four groups as follows: Aerobic Exercise + vitamin D Supplementation (AE + VitD, *n* = 10), Aerobic Exercise (AE, *n* = 10), vitamin D Supplementation (VitD, *n* = 10), and Control (C, *n* = 10). The participants in the AE + VitD and AE groups performed aerobic exercise training (running) up to 50% of the maximum heart rate, three times a week for four weeks. Participants in AE + VitD and VitD groups received 6000 IU/w vitamin D_3_ for four weeks. The participants in control group did not receive any intervention. Serum tumor necrosis factor (TNF)-α, interleukin (IL)-6, Clara cell protein (CC16), surfactant protein (SP)-D, CC16/SP-D ratio, and lung function (FEV1, FVC, and FEV1/FVC ratio) were measured before and after four weeks of intervention.

**Results:**

Serum levels of TNF-α, IL-6, and CC16 decreased significantly in AE + VitD, VitD, and AE groups after four weeks (*P* < 0.05). Serum SP-D level decreased significantly only in the AE + VitD group (*P* = 0.011). In addition, FEV1 and FVC increased significantly (*P* < 0.05) in AE + VitD and AE groups after four weeks of intervention. However, the interventions did not have a significant effect on CC16/SP-D ratio and FEV1/FVC ratio (*P* > 0.05). Furthermore, serum levels of 1,25-dihydroxyvitamin D increased significantly in AE + VitD and VitD groups (*P* < 0.05) after four weeks of intervention. However, except for TNF-α, between-group comparisons showed no significant differences in levels of IL-6, CC16, SP-D, CC16/SP-D ratio, FEV1, FVC, FEV1/FVC, and 1,25-dihydroxyvitamin D (*P* > 0.05).

**Conclusions:**

The results of present study were that aerobic exercise combined with vitamin D supplementation can reduce serum inflammatory factors and anti-inflammatory proteins and improve lung function after four weeks of intervention. Further trials with larger sample size and longer duration are suggested to confirm these results.

*Trial registration* Retrospectively registered. IRCT20180513039637N4. Registration date: 2020/10/20. URL: https://www.irct.ir/search/result?query=IRCT20180513039637N4

## Background

Evidence suggests that tobacco-related diseases and deaths are growing in the world [1]. The most common type of tobacco use is cigarette smoking (CS) [2]. Long-term cigarette smoking causes various diseases by affecting the organs of the body [3], but the most well-known effect of toxic substances of cigarettes is on the pulmonary system, which is subject to pulmonary epithelial and cell-membrane damage and altered mucosal permeability [4, 5]. These alterations cause diseases such as lung cancer and chronic obstructive pulmonary disease (COPD) [3, 6, 7]. Smoking also reduces pulmonary function by decreasing FEV_1_ and FVC [8].

Long-term cigarette smoking causes the release of inflammatory cells (e.g., macrophages, neutrophils, and lymphocytes) and inflammatory mediators (e.g., TNF-α and IL-6) from epithelial and smooth muscle cells. These inflammatory factors initiate the inflammatory process [5, 9, 10]. Moreover, toxic substances pass through the alveolar and capillaries and enter the bloodstream. These toxins are identified by immune-system receptors that trigger the inflammatory process via nuclear factor kappa B (NF-kB) in the bloodstream [5]. Nuclear factor kappa has an important role in triggering inflammation by activating the expression of genes for inflammatory factors such as IL-6 and TNF-α [11]. Also, lung epithelial cells release anti-inflammatory proteins such as Clara cell protein (CC16) and surfactant protein D (SP-D) during inflammation and disease [12, 13]. Studies have also shown that CS increases serum levels of CC16 and SP-D [13, 14]. Increasing CC16 and SP-D in serum and decreasing them in epithelial cells makes lungs more vulnerable to damage [12]. In addition, the serum CC16/SP-D ratio is a valid and sensitive marker for the diagnosis of lung epithelial cell injury [12].

Previous studies have demonstrated the non-pharmacological effect of exercise training on reducing lung diseases [5, 15]. Regular exercise can reduce inflammatory factors by activating the anti-inflammatory signaling pathway [5]. This anti-inflammatory property of exercise training improves pulmonary rehabilitation, reduces lung diseases (e.g., dyspnea, airway hyper responsiveness, exercise-induced bronchospasm, and asthma), and increases strength, endurance capacity, and quality of life [5, 15, 16]. Also, the results of Krϋger et al*.* and Toledo-Arruda et al*.* showed that aerobic exercise can reduce CS-induced inflammation [17, 18]. Furthermore, recent scientific evidence suggests that consumption of certain foods and nutrients improves lung function and reduces risks of COPD [11]. Interestingly, it has been shown that vitamin-D [defined as 1,25-dihydroxyvitamin D (1,25(OH)_2_D) > 20 ng/ml] deficiency causes lung disease and decreased lung function (FEV_1_ and FVC) [19]. Vitamin D is reported to have anti-inflammatory properties, and it can reduce CS-induced inflammation [19] by regulating the proliferation and function of immune cells through the vitamin D receptor (VDR), which is a member of the steroid hormone receptor family [20]. Agrawal et al*.* demonstrated that vitamin D supplementation can reduce inflammation by modulating the immune system [21].

To the best of our knowledge, to date, no study has examined the combined effect of aerobic exercise and vitamin D supplementation on inflammation and lung function in tobacco smokers. The purpose of the present study was to do so by examining the combination effect of aerobic exercise and vitamin D supplementation on inflammation (TNF-α, IL-6, CC16, SP-D, and CC16/SP-D ratio) and lung function (FEV1, FVC, and FEV1/FVC ratio) in 40 male smokers. We hypothesized that the interaction of aerobic exercise (running) and vitamin D supplementation (6000 IU/w) would have greater effect on the study outcomes than each one individually.

## Methods

In this experimental study, 40 healthy male smokers were recruited from Tabriz city, Iran. Inclusion criteria were as follows: (1) history of smoking cigarettes for the previous ≥ 12 months; (2) no prior history of specific diseases such as diabetes, cardiovascular and lung disease; (3) no prior history of nutritional allergies; (4) no history of medication use; (5) no prior history of using anti-inflammatory agents, β_2_-agonists and any supplements (i.e. vitamins, proteins drinks, amino acids, etc.) before or during the study; (6) no history of doing any regular exercise or physical activity in the past six months. Information on personal and family history of atopic diseases, diet, socio demographic data, and frequency and duration of cigarette smoking were collected by a general questionnaire. After reviewing the personal and family information and medical examinations, 40 healthy male smokers were selected. All participants were informed about the objectives of the study and a written consent was obtained.

The study was approved by the Research Ethics Committee of Tabriz University of Medical Sciences, Iran. Also, the present study is in accordance with the Helsinki Declaration (Ethical code: IR.TBZMED.REC.1399.727). The protocol of the study was registered in the Iranian registry of clinical trials *(*IRCT code: IRCT20180513039637N4).

Participants were randomly divided into four groups: Aerobic Exercise + vitamin D supplementation (AE + VitD, *n* = 10), Aerobic Exercise (AE, *n* = 10), vitamin D supplementation (VitD, *n* = 10), and Control (C, *n* = 10). Randomization was carried out on an individual basis using Randomizer software. Opaque and sealed envelopes were used for allocation concealment. The groups were encoded, and the codes were sealed by an independent monitor.

All participants were requested to follow their daily CS behavior and regular diet throughout the period of the study. Also, one week before the start of the study, the weight, height and body fat of the participants were measured in the laboratory. Body weight (with 10 g measurement accuracy) was measured using a digital scale (OMRON, BF: 508, Finland). Body fat was measured using an Inbody 720 body Composition Analyzer with an accuracy of 0.5% (Inbody Co. Ltd.ˏ Seoulˏ Korea). Body mass index (BMI) was calculated as weight in kilograms divided by height in meters squared. Following these measurements, participants walked to a standard racetrack where they performed the Cooper test to estimate their maximum rate of oxygen consumption (V̇O_2_ max). Participants ran as far as possible on the standard racetrack for 12 min and V̇O_2_ max was predicted based on distance covered using the following formula [22]:

$${\dot{\text{V}}\text{O}}_{2} \,\max \left( {\text{ml/kg/min}} \right) = \left( {22.351 \times {\text{distance}}\,{\text{covered}}\,{\text{in}}\,{\text{kilometers}}} \right) - 11.288$$

One week after the briefing session, the participants began consumption of vitamin D supplement and exercise. AE + VitD and AE groups performed aerobic running for four weeks (30 min for three sessions a week) on the standard racetrack [23]. For the first two weeks, AE + VitD and AE groups ran at 50–60% of their individually prescribed maximum heart rate (HR). For the second two weeks of the study, the intensity of the exercise increased to 60–70% HR max. The POLAR (T31C, Finland) HR monitors were used to estimate participants' HR to maintain prescribed intensity during exercise. The prescribed intensity was adjusted by administrator instruction during the exercise using Bluetooth headsets. The Tanaka equation [MHR = 208 − (0.7 × Age)] was used to estimate the maximum heart rate [24]. All exercise sessions were held in the morning (between 8 and 10 am). Participants in VitD and C groups were requested to perform their usual daily activities. Simultaneous with initiation of aerobic exercise, vitamin D_3_ supplementation was initiated in VitD and AE + VitD groups. Specifically, participants received 6000 IU/week (1000 IU/day = 25 µg, except Fridays) oral vitamin D_3_ in tablet form for the four weeks that followed [25]. Tablets were produced and supplied by Health Aid Company (England).

To determine the baseline and final levels (after four weeks) of IL-6, TNF-α, CC16, SP-D, and 1,25-dihydroxyvitamin D (1,25(OH)2D), blood samples were collected (from the participant's antecubital vein after 12 h of overnight fasting) 24 h before the start of the study (10 ml) and 24 h after the last aerobic exercise session (10 ml). Samples were centrifuged at 3,000 rpm for 15 min, and serum was subsequently distributed in 1.8-mL aliquots and stored at − 20 °C. Serum levels of IL-6, TNF-α, CC16, and SP-D were measured by an East Biofarm (USA) ELISA kit using the sandwich enzyme-linked immune sorbent assay method. The assay ranges for TNF-α, IL-6, CC16, and SP-D were 3–900 ng/l, 2–600 ng/l, 1–380 ng/ml, and 0.2–600 ng/ml respectively. Serum levels of 1,25-dihydroxyvitamin D were analyzed by ELISA kit using the sandwich enzyme-linked immune sorbent assay method (Euroimmun, USA). The range of assay for 1,25-dihydroxyvitamin D was 4–120 ng/ml.

FEV1 and FVC were measured before and after the study with an Easy One portable spirometer (ndd Medical Technologies, Zürich, Switzerland) following standard recommendations [26–28]. FEV1 and FVC were expressed as percentage of predicted value.

### Statistical analysis

Statistical analysis was performed using SPSS (version 22). Descriptive statistics were reported as means ± standard deviations. The normal distribution of variables was assessed with the Kolmogorov–Smirnov test. The data for the anthropometric characteristics, V̇O_2_ max, and smoking duration were analyzed using one way ANOVA test. Analysis of Covariance (ANCOVA) was used to analyze between-group differences. In the cases of significant F-ratio, Bonferroni post hoc test was used to identify the differences between groups. Paired samples t-test was used in order to comparing the baseline and 4 week-values of variables within each group. *P*-value less than 0.05 was considered to be statistically significant.

## Results

**Fig. 1:**
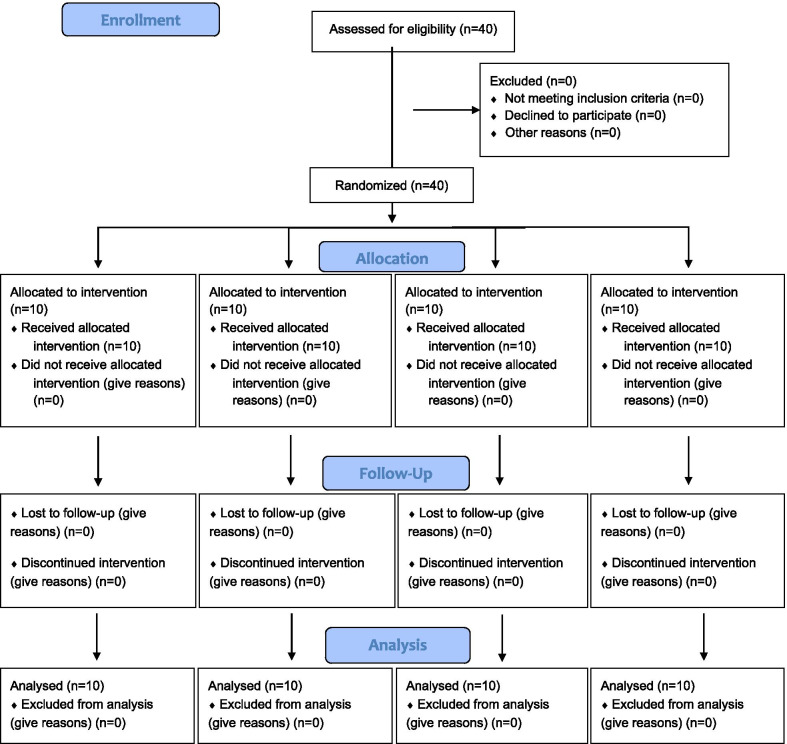
CONSORT flow diagram

A total of 40 participants were recruited and all of them completed the study (Fig. 1; Consolidated Standards of Reporting Trials (CONSORT) diagram). The anthropometric characteristics, V̇O_2_ max, and duration of cigarette consumption are provided in Table 1. Based on the results of one-way ANOVA test, there was no significant difference in anthropometric characteristics, V̇O_2_ max, and cigarette consumption between the four study groups (Table 1; *P* > 0.05).

**Table 1:** Anthropometric characteristics, V̇O_2_ max, and smoking duration in the studied groups

Variables	AE + VitD (*n* = 10)	AE (*n* = 10)	VitD (*n* = 10)	C (*n* = 10)	*P*^#^
Age, year	30.40 ± 4.08	31.30 ± 4.00	30.12 ± 3.72	31.77 ± 3.83	0.77
Height, m	1.74 ± 0.06	1.75 ± 0.07	1.74 ± 0.09	1.76 ± 0.07	0.96
Weight, kg	76.00 ± 9.00	74.00 ± 7.34	75.10 ± 9.10	76.20 ± 6.44	0.92
BMI, kg m^2^	24.90 ± 1.57	24.29 ± 0.75	25.19 ± 3.47	24.98 ± 1.70	0.80
Fat, %	14.50 ± 2.54	13.90 ± 1.85	14.90 ± 2.51	15.20 ± 1.93	0.59
V̇O_2_ max, %	41.22 ± 2.52	40.28 ± 3.25	39.30 ± 3.43	40.40 ± 2.41	0.54
History of smoking, year	6.90 ± 2.37	7.87 ± 2.62	7.65 ± 3.10	8.40 ± 5.31	0.85

Data are presented as mean ± SD

^#^One-way ANOVA

### Inflammatory factors

Table 2 provides pre- and post-intervention values for inflammatory markers, anti-inflammatory proteins, lung function, and 1,25-dihydroxyvitamin D. Following the intervention, no difference was observed for IL-6 between groups (*P* = 0.079) whereas a difference in TNF-α was present between AE + VitD and other groups (*P* = 0.001). Pre/post comparison revealed that AE + VitD, AE and VitD each experienced a significant decrease in TNF-α and IL-6 (*P* < 0.05) whereas no changes were observed in control.

**Table 2:** Baseline and Week-4 values of inflammatory markers, anti-inflammatory proteins, lung function, and 1,25-dihydroxyvitamin D

Variables	AE + VitD(*n* = 10)	AE(*n* = 10)	VitD(*n* = 10)	C(*n* = 10)	*P*^#^
TNF-α, (ng/ml^−1^)					
Baseline	73.25 ± 12.40	75.40 ± 15.05	73.99 ± 13.52	74.10 ± 10.67	0.001*
Week 4	55.05 ± 11.41^a^	66.80 ± 16.66^b^	67.70 ± 11.18^b^	74.93 ± 9.49^b^	
*P*^¥^	0.001*	0.009*	0.020*	0.394	
IL-6, (pg/ml^−1^)					
Baseline	5.87 ± 1.61	6.30 ± 2.02	5.95 ± 2.11	6.16 ± 1.39	0.079
Week 4	4.98 ± 1.37	5.93 ± 1.67	5.70 ± 1.98	6.31 ± 1.37	
P	0.004*	0.039*	0.049*	0.097	
CC16, (ng/ml^−1^)					
Baseline	7.19 ± 1.28	7.34 ± 1.07	7.24 ± 1.81	7.25 ± 1.05	0.064
Week 4	6.42 ± 1.06	7.11 ± 1.02	6.87 ± 1.08	7.34 ± 0.97	
P	0.001*	0.005*	0.025*	0.688	
SP-D, (ng/ml^−1^)					
Baseline	69.80 ± 13.70	71.83 ± 12.99	70.75 ± 10.43	71.20 ± 11.56	0.052
Week 4	62.06 ± 9.41	69.50 ± 11.10	68.40 ± 8.39	72.30 ± 11.20	
*P*	0.011*	0.085	0.084	0.240	
CC16/SP-D					
Baseline	0.104 ± 0.014	0.102 ± 0.010	0.101 ± 0.005	0.101 ± 0.009	0.969
Week 4	0.104 ± 0.107	0.102 ± 0.008	0.099 ± 0.005	0.102 ± 0.009	
*P*	0.817	0.810	0.393	0.903	
FEV_1_, %					
Baseline	87.29 ± 7.51	88.30 ± 10.54	87.55 ± 8.48	86.70 ± 11.14	0.071
Week 4	91.70 ± 5.86	91.80 ± 8.23	89.21 ± 7.85	86.44 ± 11.30	
*P*	0.001*	0.021*	0.121	0.534	
FVC, %					
Baseline	94.39 ± 7.41	94.81 ± 12.69	92.85 ± 6.93	93.15 ± 10.10	0.063
Week 4	97.97 ± 6.94	97.65 ± 9.51	93.95 ± 7.99	92.55 ± 10.73	
*P*	0.002*	0.035*	0.313	0.133	
FEV_1_/FVC, %					
Baseline	92.45 ± 2.59	93.42 ± 3.97	94.20 ± 4.08	92.89 ± 3.59	0.218
Week 4	93.78 ± 3.71	94.17 ± 3.99	95.06 ± 4.11	93.31 ± 4.22	
*P*	0.153	0.397	0.643	0.478	
1,25(OH)_2_D, (ng/ml^−1^)					
Baseline	23.90 ± 5.72	23.38 ± 6.65	24.51 ± 7.82	24.73 ± 7.60	0.085
Week 4	27.48 ± 6.85	24.15 ± 5.51	27.55 ± 8.05	24.64 ± 7.18	
*P*	0.002*	0.237	0.001*	0.924	

Data are presented as mean ± SD, #ANCOVA (Analysis of Covariance). Bonferroni adjustment for multiple comparison, different letters means statistically difference with Bonferroni adjustment

^¥^Paired sample T-test, * < 0.05, There is significant difference

### Anti-inflammatory proteins

Following the intervention, there were no significant differences between groups for CC16, SP-D or CC16/SP-D (*P* > 0.05; Table 2). Pre/post comparison revealed that AE + VitD (*P* = 0.001), AE (*P* = 0.005), and VitD (*P* = 0.025) each experienced a significant decrease in CC16, although only AE + VitD experienced a significant decrease in SP-D (*P* = 0.011). Conversely, CC16/SP-D was not altered by the intervention in any of the four groups (*P* > 0.05).

### Lung function

Following the intervention, there were no significant differences between groups for FEV1, FVC or FEV1/FVC (*P* > 0.05; Table 2). Pre/post comparison revealed that AE + VitD and AE each experienced a significant increase in FEV1 and FVC (*P* < 0.05) while FEV1/FVC was not altered by the intervention in any of the four groups (*P* > 0.05).

### Serum 1,25-dihydroxyvitamin D concentration

Following the intervention, there was no significant difference between groups for 1,25-dihydroxyvitamin D (*P* = 0.085; Table 2). Pre/post comparison revealed that AE + VitD and VitD each experienced a significant increase in 1,25-dihydroxyvitamin D (*P* = 0.002 and *P* = 0.001, respectively).

## Discussion

The results of this study demonstrated that TNF-α, IL-6, and CC16 were reduced significantly in all three intervention groups in this study. Confirming the study hypothesis, between-group comparisons showed that only the combination of aerobic exercise and supplementation brought a reduction in TNF-α in comparison to other groups and SP-D decreased significantly just in AE + VitD group. However, in contrast to study hypothesis, exercise without supplementation was sufficient for improving lung function in smokers.

Based on the results, AE + VitD, AE, and VitD each experienced a significant decrease in TNF-α and IL-6. Nevertheless, only the combination of aerobic exercise and vitamin D supplementation brought a reduction in TNF-α in comparison to other groups. This result confirmed that collectively, aerobic exercise training and vitamin D supplementation exert a synergistic effect on decreasing inflammation.

To the best of our knowledge, the present study is the first one to analyze the combined effect of exercise training and vitamin D supplementation on inflammation. Previous findings suggested that physical activity [7, 18] and vitamin D supplementation [19, 20] alone can reduce inflammatory cytokines. Exercise training moderates inflammatory process by activating anti-inflammatory signaling pathways [5]. Exercise training inserts its anti-inflammatory properties by increasing systemic levels of anti-inflammatory cytokines such as IL-1RA, and prevents the secretion of pro-inflammatory factors [5]. Prolonged exercise also plays an important role in reducing the production of the pro-inflammatory factors by decreasing expression of Toll-like receptors (TLRs) on monocytes [5]. In addition, vitamin D supplementation reduces inflammation by inhibiting the proliferation of T-cells and the production of cytokines [20].

According to the results of our study, aerobic exercise alone, vitamin D supplementation alone and the two combined each reduced CC16 after four weeks; however, the combination of aerobic exercise and vitamin D supplementation had greater effect on SP-D than each one individually and only AE + VitD group experienced a considerable decrease in SP-D after four weeks. Therefore, the combination of aerobic exercise and vitamin D supplementation will have greater effect on anti-inflammatory proteins than each one alone.

There is a limited research on the effect of aerobic exercise and vitamin D supplementation (separately and simultaneously) on serum CC16 and SP-D levels. However, in one study, Moazami et al*.* reported that aerobic exercise decreased serum CC16 [29], which is used as a marker of distal lung epithelial damage [30]. Although biological function of CC16 has not been completely clarified, it seems that it can also have direct anti-inflammatory properties through inhibiting the enzyme phospholipase A_2_ and the protein prostaglandin D_2_ release. These are each involved in the arachidonic acid inflammatory cascade [31]. Also, SP-D is a protein in pulmonary host defense that is released from the epithelial cells of the lungs to interact with alveolar macrophages and a variety of microorganisms [13, 32, 33].

Based on the results of present study, aerobic exercise alone and the combination of aerobic exercise and vitamin D supplementation significantly increased FEV_1_ and FVC after four weeks in male smokers. Therefore, exercise without supplementation appears to be sufficient for improving lung function. To the best of our knowledge, the combined effect of vitamin D supplementation and aerobic exercise on lung function has not been studied previously. According to the results of previous studies, physical activity improves lung function [34–36]. Garcia et al*.* reported that moderate to high levels of regular exercise increases lung function in smokers [37]. It seems that regular exercise prevents lung-function decline by reducing inflammatory markers and oxidative stress. Also, exercise has a positive effect on respiratory muscle strength [37]. In contrast with previous studies [19], in this study, vitamin D supplementation had no effect on improving lung function. Vitamin D sufficiency protects lung function against the inflammatory and oxidative effects of smoking [38]. We speculate that four weeks of supplementation was not enough to see the positive effects on lung function. Consequently, it is possible that studies with longer durations are needed.

### Limitations

The results of this study should be interpreted considering the limitations. In this study, the sample size was small and the duration of study was short. Bigger sample size would increase the statistical power of the study and longer duration may result in the interventions exerting a greater effect on the study parameters. Also, we did not measure the participants' sunlight exposure in this study. Our skin has cells that produce vitamin D when exposed to the sun; therefore, such exposure (which we did not control for) might have affected [39].

## Conclusions

In conclusion, the results of present study demonstrated that aerobic exercise (running) and vitamin D supplementation (6000 IU/w) alone and the combination of aerobic exercise and vitamin D supplementation remarkably reduced TNF-α, IL-6, and CC16. In addition, aerobic exercise alone and the combination of aerobic exercise and vitamin D supplementation significantly increased FEV1 and FVC. In contrast with independent effects, the combination of an aerobic exercise with vitamin D supplementation had significant effect on TNF-α and SP-D in male smokers. Further larger trials with higher sample size, on female smokers, and longer duration are suggested to confirm these results.

## Data Availability

If requested, it will be available.

## Abbreviations

AE + VitD: Aerobic exercise + vitamin D supplementation
AE: Aerobic exercise
VitD: Vitamin D supplementation
C: Control
HR: Heart rate
TNF-α: Tumor Necrosis factor-α
IL-6: Interleukin-6
CC16: Clara cell protein
SP-D: Surfactant protein-D
FEV_1_: Forced expiratory volume in one second
FVC: Forced vital capacity
CS: Cigarette smoking
LTCS: Long term cigarette smoking
COPD: Chronic obstructive pulmonary disease
1,25(OH)_2_D: 1,25-Dihydroxyvitamin D
VDR: Vitamin D Receptor
BMI: Body mass index
V̇O_2_ max: Maximum rate of oxygen consumption
ANOVA: Analysis of variance
ANCOVA: Analysis of covariance
TLRs: Toll-like receptors
